# Animal Models for Swine Influenza Virus Research: Pathology, Viral Dynamics, and Immune Responses

**DOI:** 10.3390/v18030344

**Published:** 2026-03-11

**Authors:** Jingyu Zhang, Shuaiyu Jiang, Yupeng Fang, Jiahong Feng, Wenqing Zhang, Xiaoqing Zhang, Jie Zhang

**Affiliations:** Hebei Key Laboratory of Preventive Veterinary Medicine, Hebei Normal University of Science and Technology, Qinhuangdao 066000, China; 18831218061@163.com (J.Z.); 15630615258@163.com (S.J.); fangyp@hevttc.edu.cn (Y.F.); 17302590392@163.com (J.F.); 19861911507@163.com (W.Z.)

**Keywords:** swine influenza virus (SIV), animal models, pathogenesis, viral transmission, immune response, vaccine evaluation, multi-model strategy

## Abstract

Swine influenza virus (SIV) continues to evolve and possesses notable zoonotic potential, making it an important respiratory pathogen of concern for both the global swine industry and public health. Owing to antigenic drift, genetic reassortment, and regional lineage diversity, vaccine efficacy against SIV shows marked variability across different epidemiological contexts. Therefore, establishing appropriate animal models to dissect its pathogenic mechanisms, transmission characteristics, and immune response patterns is of critical importance. This review systematically summarises the animal models commonly used in SIV research, including mice, ferrets, guinea pigs, pigs, and non-human primates, and provides an integrated analysis across three core dimensions: pathological manifestations, viral replication kinetics, and immune architecture. The evidence indicates that substantial inter-model differences exist in pulmonary lesion distribution, transmission efficiency, mucosal immune development, and cellular immune complexity, which in turn define their functional roles in mechanistic studies, transmission research, and vaccine evaluation. Building on this framework, this review further emphasises the value of a tiered, multi-model strategy in SIV research. In vitro systems and mouse models are well suited for early mechanistic exploration and preliminary vaccine screening; ferret and guinea pig models facilitate the evaluation of transmission dynamics; and the pig model, as the natural host system, remains the critical platform for confirming protective efficacy, identifying potential immunopathological risks, and assessing translational relevance. Importantly, the potential occurrence of vaccine-associated enhanced respiratory disease under antigen-mismatched conditions highlights the need to evaluate both protective performance and immunological safety during vaccine development. Overall, rational integration of evidence across multiple models, anchored to the natural host, will improve the predictability and translational reliability of SIV vaccine research.

## 1. Introduction

Swine influenza virus (SIV) belongs to the family *Orthomyxoviridae* and is one of the major respiratory pathogens affecting the global swine industry. The viral genome consists of segmented negative-sense RNA, which readily undergoes genetic reassortment during co-infection of a host. Because porcine respiratory epithelial cells express both α-2,3 and α-2,6 sialic acid receptors, pigs are susceptible to both avian- and human-origin influenza viruses, making them an important “mixing vessel” for cross-species transmission and viral reassortment [1]. The 2009 pandemic H1N1 influenza virus originated from a triple-reassortant swine strain, highlighting the potential public health risk posed by SIV in emerging and re-emerging infectious diseases [2].

It is important to note that, although human influenza models have generated extensive mechanistic insights, their findings cannot be directly extrapolated to the swine influenza context. First, humans and pigs differ in respiratory tract architecture, pulmonary immune microenvironment, and the distribution of tissue-resident T cells [3]. Second, systematic interspecies differences exist in innate immune receptor expression profiles, inflammatory response intensity, and viral clearance kinetics. In addition, transmission dynamics within swine populations—such as herd density, housing conditions, and co-infection patterns—differ substantially from those in human populations [4]. Therefore, directly applying conclusions from human influenza studies to SIV research may lead to misinterpretation of immune protection mechanisms or vaccine efficacy.

At present, inactivated and live attenuated vaccines are widely used in swine populations. However, vaccine effectiveness varies markedly among regions and herds, largely due to antigenic drift, genetic reassortment, and region-specific lineage evolution of SIV [5]. Moreover, natural infection and vaccination differ significantly in their ability to induce mucosal immunity, local immune memory, and tissue-resident T cell responses [6], making accurate evaluation of cross-protective immunity an urgent challenge.

Accordingly, the establishment of appropriate animal models is essential for investigating SIV pathogenesis, host immune responses, transmission characteristics, and vaccine efficacy [7]. However, no single animal model fully recapitulates the pathological and immunological features of natural infection in pigs. Mouse models are genetically tractable and suitable for mechanistic studies but poorly reproduce porcine pulmonary mucosal immunity. Ferret and guinea pig models are valuable for transmission research but differ substantially from pigs in immune system architecture. Non-human primates share closer immune kinetics with humans but are limited by high cost and ethical constraints. In contrast, the pig model most faithfully reflects immune responses and disease course in the natural host, yet its use is restricted by experimental complexity and expense.

This leads to a central issue in current research: systematic differences in immune responses among animal models can directly influence the evaluation of vaccine efficacy and the interpretation of immune mechanisms.

Therefore, a comprehensive understanding of inter-model differences in innate immunity, humoral immunity, mucosal immunity, and tissue-resident T cell formation is critical for rational model selection and for improving the interpretability and translational relevance of vaccine studies. This review systematically compares commonly used SIV animal models from the perspective of immune response heterogeneity and discusses their implications for vaccine development and applied research.

## 2. Biological Characteristics and Epidemiological Shifts in SIV

### 2.1. Genome Architecture of SIV and Host Receptor Characteristics

Swine influenza virus (SIV) is an influenza A virus (IAV) with an eight-segmented, negative-sense RNA genome. Each segment encodes distinct structural or functional proteins, including haemagglutinin (HA), neuraminidase (NA), and nucleoprotein (NP) [3]. Porcine respiratory epithelial cells express both α-2,3- and α-2,6-linked sialic acid receptors, enabling pigs to be infected by influenza viruses of avian as well as human origin; consequently, pigs are often regarded as a “mixing vessel” for influenza virus reassortment. This receptor distribution provides a molecular basis for co-infection by viruses from different sources and subsequent genome segment reassortment, thereby creating key preconditions for the emergence and onward spread of novel influenza subtypes.

### 2.2. Epidemiological Dynamics of Swine Influenza Virus

SIV circulates predominantly as the H1N1, H1N2, and H3N2 subtypes, and the molecular evolutionary lineages and antigenic properties of circulating strains continue to change across regions worldwide. Following the 2009 influenza A(H1N1) pandemic, triple-reassortant viruses spread rapidly on a global scale and displaced multiple previously prevailing lineages [8]. In recent years, alongside increasing inter-regional movements of swine and the globalisation of pig production systems, the H1N1, H1N2, and H3N2 subtypes have remained in sustained circulation in China and globally. Ongoing emergence of novel reassortant genotypes and antigenic drift variants has further complicated vaccine strain selection, resulting in progressively greater challenges in matching vaccine strains to field viruses [9,10,11,12,13,14,15,16].

## 3. Establishment of SIV Animal Infection Models and Evaluation Parameters

The primary objective in establishing animal models of swine influenza virus (SIV) infection is to reproduce, to the greatest extent possible, the infection kinetics, histopathological progression, and immune-response trajectories observed in the natural host [17]. The quality of model construction directly determines the practical relevance of experimental outcomes. This section describes three key dimensions: the critical parameters for simulating natural infection, the evaluation endpoints, and the applicability of different animal models.

### 3.1. Key Parameters for Modelling Natural Infection

To generate experimental data with clinical relevance, the construction of animal models must carefully control infection routes and inoculation doses in order to minimise artefacts introduced by conventional experimental approaches [18].

(1) In swine populations, the natural transmission of SIV mainly occurs through aerosols, inhalation of respiratory droplets, and short-range contact. Although traditional high-dose intranasal inoculation or intratracheal administration can ensure high infection rates, these approaches often result in direct viral deposition in the deep lower respiratory tract and can induce non-physiological, acute severe pneumonia. Such artificial exposure may obscure the early steps of viral adhesion to and invasion of the respiratory mucosa. Therefore, to more accurately mimic natural infection, an ideal model should prioritise aerosol nebulization or environmentally simulated contact exposure [19]. These approaches better reflect low-dose, multi-site exposure and can more accurately capture the initial colonisation pattern along the respiratory epithelium.

(2) Concordance of viral replication and disease kinetics: A qualified model should reproduce, to a reasonable extent, the temporal dynamics observed during natural infection in pigs. Under natural conditions, infection typically follows a defined timeline: clinical signs emerge at approximately 2–3 days post infection (dpi), pulmonary lesions and viral shedding peak at 5–7 dpi, and viral clearance is largely completed by 10–14 dpi. If viral clearance occurs too rapidly (as seen in some non-adapted mouse strains) or if infection persists excessively, misinterpretation of vaccine durability or antiviral half-life may occur [20].

(3) Comprehensiveness of immune responses: Protective immunity against SIV depends on a coordinated network of systemic and mucosal immunity. An appropriate model must be capable of inducing and detecting key immunological readouts, including humoral immunity (serum HI and neutralising antibodies), mucosal immunity (nasal and pulmonary sIgA), and cellular immunity (Th1-associated cytokines and cytotoxic T lymphocyte responses) [21,22,23]. Reliance on a single serological parameter is often insufficient to accurately evaluate vaccines targeting this predominantly mucosal pathogen.

To avoid overgeneralization, it should be emphasised that peak viral load and replication kinetics are not fixed parameters but vary substantially depending on viral strain, inoculation dose, infection route, and host species. Highly pathogenic or host-adapted strains may replicate more rapidly and reach peak viral loads earlier, whereas low-dose aerosol exposure typically results in delayed replication and more spatially restricted infection. Likewise, compared with contact-based transmission models, intratracheal inoculation tends to artificially synchronise lower respiratory tract infection. Therefore, these variables should be carefully controlled and explicitly reported when interpreting cross-model comparisons.

### 3.2. Evaluation Parameters for Natural Infection-Mimicking Models

A standardised SIV animal model should demonstrate high stability and reproducibility across three key dimensions: pathological injury, viral replication kinetics, and immune phenotype [18].

(1) Pathological characteristics

Model animals should exhibit gross and microscopic lesions comparable to those observed in naturally infected pigs [24]. Gross pathology: Typical findings include well-demarcated purple-red consolidation of affected lung lobes, most commonly involving the cardiac and apical lobes, often accompanied by thickening of the alveolar septa. Histopathology: Characteristic necrotizing bronchitis should be evident, including necrosis and sloughing of tracheal/bronchial epithelial cells, neutrophilic exudation, and prominent peribronchiolar lymphocytic cuffing.

(2) Viral replication kinetics

The spatiotemporal distribution of viral load is a critical indicator of model sensitivity [25]. In a standard SIV model, viral titres typically peak at 1–2 days post infection, leading to viral pneumonia, and viral loads in lung tissue should be significantly higher than those in the upper respiratory tract. In addition, the model should exhibit differential replication efficiency among major subtypes (H1N1, H1N2, H3N2), thereby accurately reflecting differences in pathogenicity and immune evasion among circulating strains.

(3) Immune response profiling

Evaluation metrics should encompass local, systemic, and cellular immunity. For local immunity, virus-specific IgA can be detected in the nasal cavity (nasal wash/swab) and lower respiratory tract (bronchoalveolar lavage, BAL) following infection or immunisation in pigs, with IgA serving as a key isotype at mucosal surfaces [21]. For systemic immunity, serum hemagglutination inhibition (HI) antibody titres are commonly used in swine influenza studies and vaccine assessments as indicators of humoral responses [26]. For cellular immunity, CD4^+^ and CD8^+^ T cell responses can be evaluated in blood, BAL, and tracheobronchial lymph nodes, together with key cytokine expression profiles to characterise immune phenotypes and their correlation with protection [27].

### 3.3. Core Advantages and Limitations of Model Selection

Given the complexity of SIV infection and the fact that no single model can fully recapitulate the pathological features observed in pigs, the selection of an appropriate animal model requires consideration of multiple factors. A tiered selection strategy is therefore essential, and models should be chosen according to their alignment with the research objective, biological relevance to the natural host, and practical experimental constraints [28]. The major advantages and limitations of commonly used animal models in SIV research are summarized below, with detailed comparisons presented in Table 1.

(1) Mouse model: preferred platform for fundamental mechanistic studies

Mice possess a well-defined genetic background, abundant immunological reagents, and high experimental tractability [20]. Their primary advantage lies in the ability to dissect virus–host interactions through gene knockout and transgenic approaches, making them a core platform for early drug screening and mechanistic investigations [20]. However, it should be noted that not all swine-origin H1N1 strains replicate efficiently in mice without prior adaptation. Some strains exhibit cross-species replication barriers and often require serial passaging to acquire adaptive mutations for stable infection. This adaptation process may alter viral pathogenicity and thus deviate from the behaviour observed in the natural host. In addition, the distribution of respiratory epithelial receptors in mice differs from that in pigs, resulting in lower susceptibility to natural infection. Mouse lung pathology also poorly mimics the diffuse lesions seen in pigs, and aerosol transmission is relatively inefficient, limiting its utility for modelling natural transmission dynamics.

(2) Ferret model: gold standard for influenza transmission studies

Ferrets exhibit respiratory receptor distributions highly similar to those of humans and pigs and are highly susceptible to influenza virus replication and aerosol transmission. Therefore, they are widely regarded as an ideal model for studying transmission dynamics, viral adaptive evolution (e.g., antigenic drift and reassortment), and cross-species transmission potential [29]. Despite its unique value in evaluating viral spread and replication efficiency, the ferret model is constrained by high costs, limited availability of immunological reagents, and relatively small sample sizes. These limitations restrict its broad application in vaccine immunology and necessitate complementary use with other models [30].

(3) Guinea pig model: an intermediate option for drug screening and transmission studies

Guinea pigs display efficient aerosol transmission characteristics and are suitable for investigating viral spread and environmental stability [31]. However, guinea pigs generally lack typical influenza-like clinical signs, and their pulmonary pathological changes are relatively mild, preventing full recapitulation of the natural infection process in pigs. In addition, the limited availability of immunological reagents in this model constrains in-depth cellular immune profiling and mechanistic studies. Consequently, the guinea pig model is more appropriate as a complementary system for transmission dynamics alongside the ferret model rather than for comprehensive vaccine immunogenicity evaluation [32,33].

(4) Pig model: the gold standard closest to the natural host

As the natural host of SIV, pigs represent the gold standard model that most closely reflects natural infection. Pigs can faithfully reproduce clinical manifestations such as fever, lung injury, and respiratory distress, and their viral replication kinetics and host immune responses closely mirror those observed in field infections. Therefore, the pig model is the preferred system for evaluating vaccine protective efficacy, clinical outcomes, viral clearance, and potential immunopathological effects [18]. However, the pig model is associated with high experimental costs, strict biosecurity requirements, and longer study durations, which reduce its flexibility for large-scale screening and early mechanistic exploration compared with mice and ferrets.

(5) Non-human primate models: advanced systems for cross-species risk assessment

Non-human primates (e.g., rhesus macaques and cynomolgus macaques) possess immune systems highly similar to humans and are therefore valuable for assessing the cross-species transmission potential of SIV genotypes and their possible risks to human health [34,35]. Nevertheless, the high cost, stringent ethical oversight, and limited sample sizes restrict their use primarily to high-risk genotypes or advanced studies with direct public health relevance.

In addition to these in vivo systems, in vitro and ex vivo models provide important complementary tools. For example, primary porcine respiratory epithelial cells and precision-cut lung slices can simulate local host responses and tissue-level infection dynamics within a shorter experimental timeframe, serving as an intermediate bridge between in vitro mechanisms and in vivo models [36]; Cell lines such as MDCK and PK-15 are widely used for viral replication kinetics and antiviral drug screening, substantially reducing reliance on animal experiments [37]. Although these complementary systems cannot replace animal infection models, they provide efficient and controllable platforms for mechanistic exploration, virus–host interaction studies, and early therapeutic evaluation.

At present, no single animal model can fully recapitulate the complete biological characteristics of pigs as the natural host of swine influenza virus (SIV). The strengths and limitations of each animal model vary depending on the specific research objective. Therefore, reliance on a single model is insufficient to comprehensively cover key aspects of SIV research, including viral replication kinetics, histopathological changes, immune regulatory mechanisms, and vaccine immunogenicity.

To ensure both the comprehensiveness and accuracy of experimental findings, selecting the most appropriate animal model based on the study objective—or adopting a multi-model validation strategy—has become a critical approach in SIV pathogenesis research and vaccine evaluation. This section provides the methodological framework for the subsequent systematic comparisons of pathology, viral dynamics, and immune responses.

## 4. Animal Models of SIV Infection: Differences in Pathological Changes and Immune Responses

The strong interspecies transmission potential of SIV, together with the high heterogeneity among viral strains, results in pronounced differences across animal models in their ability to mirror the natural course of infection. These differences are particularly evident in patterns of tissue injury, anatomical sites of viral replication, magnitude of immune responses, and capacity for aerosol transmission, with each model presenting distinct strengths and limitations [38,39,40]. This section compares, in a model-by-model manner, the pathological manifestations and immunological features of the five most commonly used systems in current research—mice, ferrets, guinea pigs, pigs, and non-human primates—to inform subsequent vaccine evaluation and mechanistic studies of pathogenesis.

### 4.1. Mouse Models: High Genetic Manipulability but Substantial Immunological Limitations

As a classical small-animal experimental system, mice are widely used to investigate influenza virus pathogenesis and host immune responses [41,42,43]. Numerous studies have shown that swine-origin H1N1 viruses or recombinant influenza viruses from different sources can replicate efficiently in mice and reproducibly induce acute respiratory disease dominated by pulmonary injury, with relatively consistent pathological manifestations [44]. Following infection, gross lung lesions are commonly observed, characterised by varying degrees of pulmonary consolidation and focal or diffuse pneumonic foci; lesion severity correlates closely with viral replication levels in the lungs and with lethality in severe cases [45].

Histopathological examination indicates that murine influenza infection most frequently manifests as acute bronchitis or bronchiolitis accompanied by acute interstitial pneumonia (or bronchointerstitial pneumonia–like changes). Infected lungs show epithelial damage and sloughing within the airways, with cellular debris and exudates present in some lumina. Thickening of alveolar septa and prominent infiltration of inflammatory cells into alveolar spaces and the interstitium are also evident, typically dominated by neutrophils and macrophages, and may be accompanied by pulmonary oedema, haemorrhage, and atelectasis. Notably, infection with highly pathogenic or lethal strains or recombinant viruses often produces more pronounced necrotising bronchitis or bronchiolitis, together with severe alveolitis and pulmonary oedema as dominant pathological features. By contrast, infection with certain swine-derived 2009/H1N1 isolates tends to produce lesions characterised primarily by acute interstitial pneumonia, alveolar wall thickening, inflammatory infiltrates, and haemorrhage or atelectasis, which commonly become prominent several days post infection (e.g., 3–5 days post infection).

At the immunological level, influenza virus infection in mice rapidly induces acute inflammatory responses driven largely by innate immune activation. During the early stages of infection, type I interferon responses in the lung—particularly IFN-β—are markedly upregulated, accompanied by increased expression of pro-inflammatory cytokines such as TNF-α and multiple chemokines, including MIP-1α and MIP-2 [46,47,48,49]. Production of these mediators correlates closely with pulmonary viral loads and constitutes a major molecular basis for the development of acute inflammation and immunopathological lung injury. Concomitantly, large numbers of neutrophils are rapidly recruited to lung tissue and alveolar spaces and, together with activated alveolar macrophages, contribute to inflammatory amplification and tissue damage, forming the principal cellular components of early antiviral immunity and immunopathology in murine influenza models.

Multiple murine influenza studies further indicate that adaptive immunity progressively contributes to viral clearance as infection advances. During the mid to late stages of infection, virus-specific immune cell responses and cytokine changes can be detected in lung tissue and bronchoalveolar lavage fluid, while certain anti-inflammatory or immunoregulatory mediators, such as IL-10, are upregulated at specific time points, suggesting that negative-feedback regulatory mechanisms are gradually engaged following intense inflammatory responses [50].

**Figure 1:**
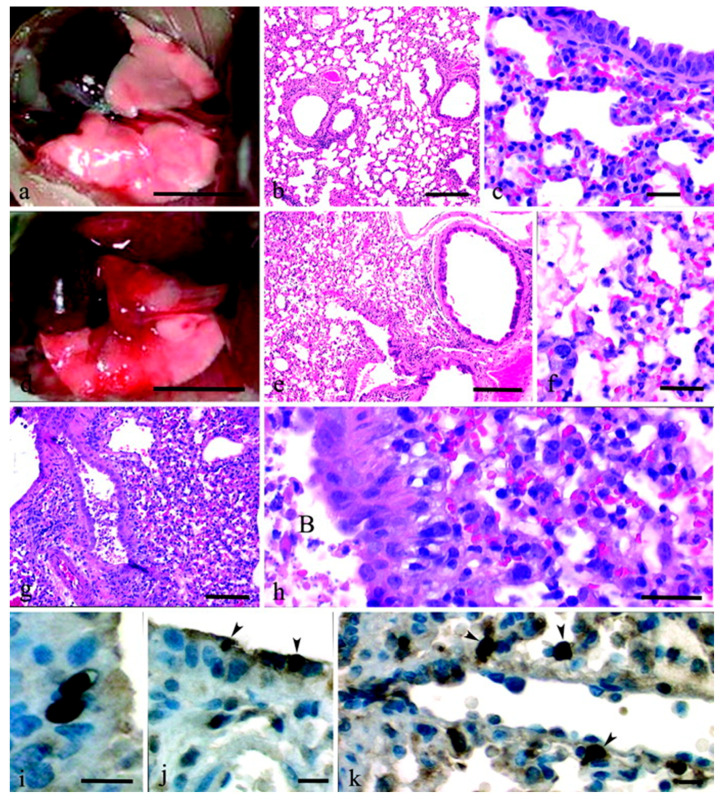
Pulmonary pathological changes in mice following intranasal inoculation with different recombinant influenza viruses. (**a**) At day 5 post inoculation with the Tx/91 virus, the gross appearance of the lungs was largely normal, with only focal pneumonia observed in the left lung lobe. (**b**,**c**) At day 5 after inoculation with the Tx/91 virus, lung tissues showed predominantly mild peribronchiolar inflammation and alveolitis dominated by histiocytes, accompanied by mild alterations in alveolar architecture. (**d**) At day 5 after inoculation with the 1918 HA/NA:Tx/91 recombinant virus, extensive pneumonic lesions involving multiple lung lobes were evident. (**e**,**f**) At day 5 after inoculation with the 1918 HA/NA:Tx/91 recombinant virus, lung tissues exhibited diffuse, severe alveolitis, with necrotising bronchitis in regions adjacent to bronchioles, accompanied by alveolar oedema. (**g**,**h**) At day 5 after inoculation with the 1918 HA/NA:WSN recombinant virus, severe necrotising bronchitis and alveolitis were observed, with abundant necrotic debris within the bronchial lumen and marked inflammatory cell infiltration in the alveolar spaces. (**i**,**j**,**k**) Immunohistochemical staining demonstrated viral antigen-positive signals in bronchial epithelial cells and alveolar macrophages in lung tissues following infection with the 1918 HA/NA:Tx/91 recombinant virus.

Overall, murine influenza models are characterised by pronounced inflammatory reactions and innate immune cell infiltration accompanied by the participation of immunoregulatory pathways, providing an important experimental basis for dissecting influenza virus pathogenic mechanisms [51]. Representative gross and histopathological lung lesions observed in mice following influenza virus infection are shown in Figure 1 [52], which systematically illustrates typical patterns of pulmonary injury induced by different viral strains in this model.

### 4.2. Ferret Models: Human-like Transmission Patterns but Incomplete Pathology

Ferrets exhibit respiratory tract sialic acid receptor distributions highly similar to those of humans and are therefore among the most widely used small-animal models for studying the transmissibility and pathogenicity of human influenza viruses [53]. Following infection with swine-origin H1N1 or the 2009 pandemic H1N1 (pH1N1) virus, ferrets typically develop upper respiratory tract signs, including fever, sneezing, coughing, rhinorrhoea, and substantial nasal viral shedding [54]. Previous studies have further demonstrated that, in combined nasal and tracheal infection models, H5N1, pH1N1, and seasonal influenza viruses exhibit markedly different respiratory pathogenic dynamics, with characteristic variations in the timing of lesion development, the intensity of inflammation, and the anatomical sites involved [55].

Systematic comparisons of three representative influenza viruses—seasonal H3N2, 2009 pH1N1, and highly pathogenic H5N1—have shown distinct patterns of tissue tropism and lesion severity within the ferret respiratory tract. Replication of H3N2 is most restricted, occurring primarily in the nasal epithelium and causing only mild mucosal injury; pH1N1 replicates throughout the respiratory tract and induces moderate bronchopneumonia, whereas H5N1 infection is concentrated in alveolar regions, resulting in the most severe lower respiratory tract damage.

**Figure 2:**
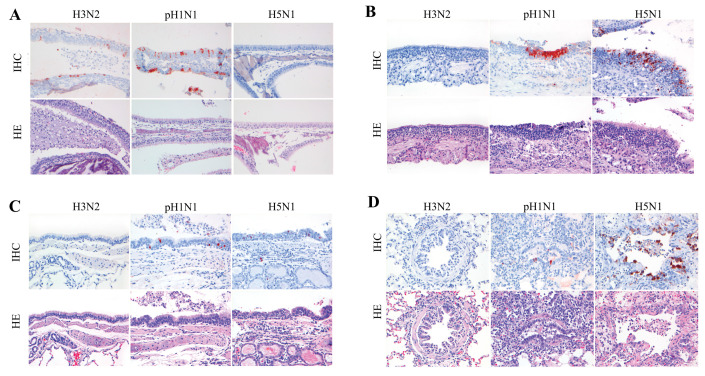
Histopathological changes in respiratory tract tissues of ferrets at 4 days post infection following intranasal inoculation with different influenza virus subtypes. (**A**) Nasal epithelium: In ferrets infected with H3N2, the nasal mucosal architecture remained largely intact, with only sparse inflammatory cell infiltration and very few antigen-positive cells detected. In pH1N1-infected animals, evident epithelial damage and more extensive antigen-positive areas were observed. Following H5N1 infection, epithelial necrosis and inflammatory responses were more pronounced, with the strongest antigen signals. (**B**) Bronchial epithelium: H3N2 infection produced no obvious antigen detection in bronchial segments and only mild inflammation. In contrast, pH1N1 infection caused focal necrosis of bronchial epithelial cells accompanied by moderate inflammatory infiltration and the presence of antigen-positive cells, whereas H5N1 induced more severe bronchial injury, with abundant viral antigen distributed in the epithelium and surrounding tissues. (**C**) Bronchiolar region: Lesions associated with H3N2 were mild, with little or no detectable viral antigen. After pH1N1 infection, prominent antigen-positive signals were observed in the bronchiolar epithelium together with marked inflammatory cell infiltration. H5N1 infection produced similar but more extensive damage, with greater inflammatory severity. (**D**) Alveolar region: In H3N2-infected ferrets, alveolar structures were largely preserved, with only mild focal inflammation. pH1N1 infection resulted in moderate alveolitis with conspicuous inflammatory infiltration and detectable antigen-positive cells. H5N1 caused the most severe alveolar injury, characterised by widespread alveolitis, necrosis, and haemorrhage, with abundant viral antigens distributed throughout the affected areas.

Histological sections obtained at 4 days post infection following inoculation with equivalent viral doses clearly illustrate these differences. In H3N2-infected ferrets, only a small number of antigen-positive cells are detected in the nasal cavity, with minimal bronchial and alveolar inflammation. In contrast, pH1N1 infection is associated with more prominent viral antigen expression and inflammatory infiltration in bronchial epithelium, bronchioles, and portions of the alveoli, whereas H5N1 viral antigen is widely distributed in the lower respiratory tract, accompanied by pronounced alveolitis, necrosis, and haemorrhage. These pathological changes correspond closely to the anatomical distribution of viral antigen and represent typical manifestations of virus-specific pathogenic features in the ferret model. As illustrated in Figure 2, this study provides a schematic summary of the spatiotemporal lesion patterns induced by the three representative strains in the ferret respiratory trac.

From an immunological perspective, ferrets typically develop detectable humoral immune responses, including haemagglutination inhibition and neutralising antibodies, approximately one week after natural influenza virus infection [56]. Concurrently, cellular immune activity characterised by IFN-γ-associated responses can be observed in infected respiratory tissues [57]. Overall, the kinetics of antibody production and patterns of local cellular immunity in ferrets closely resemble those observed in humans, underscoring their value for investigating influenza infection and immune responses [58].

Notably, repeated infection or prior immunisation in ferrets can result in immune imprinting effects similar to those described in humans, whereby exposure history substantially alters subsequent antibody specificity, cross-reactivity, and protective efficacy following reinfection or vaccination [59,60]. These findings indicate that primary exposure to a given viral strain can durably shape immune response patterns against heterologous viruses, making ferrets a valuable model for studying influenza immune memory and cross-protection.

### 4.3. Guinea Pig Models: Specialised Systems for Airborne Transmission Studies

**Figure 3:**
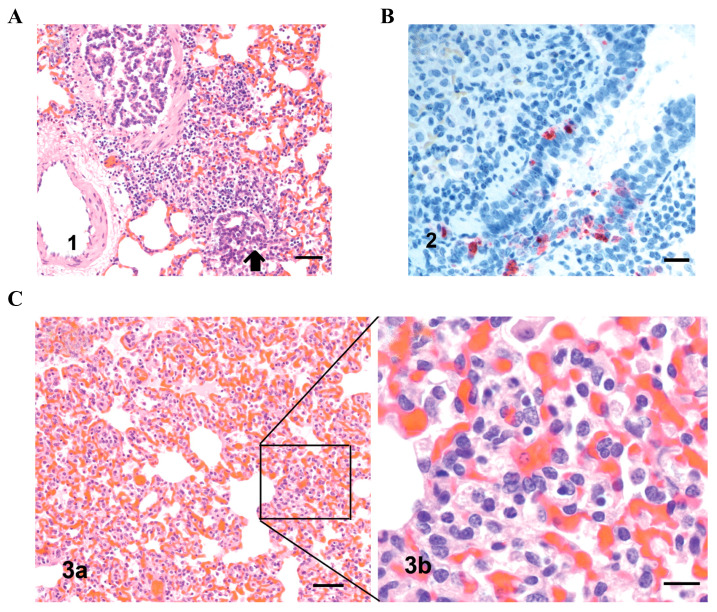
Pulmonary pathology and viral antigen distribution in guinea pigs at 5 days post infection following intranasal inoculation with the VN/04 influenza virus. Representative histopathological and immunohistochemical changes in guinea pig lung tissues at 5 days post infection (5 dpi) after intranasal inoculation with the VN/04 influenza virus are shown. (**A**) H&E staining revealed moderate lymphocytic infiltration surrounding bronchioles, accompanied by focal necrosis of the bronchiolar mucosal epithelium (arrow). Scale bar = 50 μm. (**B**) Immunohistochemical staining demonstrated that influenza virus antigen was predominantly localised in bronchiolar epithelial cells. Detection was performed using a biotin–streptavidin complex, with haematoxylin counterstaining. Scale bar = 25 μm. (**C3a**) H&E staining showed congestion and oedema within alveolar walls and alveolar spaces. Scale bar = 50 μm. (**C3b**) H&E staining revealed interstitial pneumonia characterised by infiltration of lymphocytes and histiocytes, together with histiocytic alveolitis. Scale bar = 15 μm.

Guinea pigs are highly susceptible to a wide range of human-, swine-, and avian-origin influenza viruses, yet typically develop only mild clinical signs, most often limited to transient weight loss and reduced activity [32,61,62,63]. In this context, guinea pig models show relatively limited sensitivity for assessing virulence, and pulmonary pathology is usually mild to moderate in severity. As illustrated in Figure 3, histological examination following infection commonly reveals bronchointerstitial pneumonia, mild degeneration of alveolar epithelial cells, interstitial oedema, and modest inflammatory cell infiltration dominated by lymphocytes and histiocytes, whereas extensive haemorrhagic pneumonia or classical diffuse alveolar damage is uncommon in this model.

From an immunological perspective, guinea pigs often mount relatively effective innate immune barriers following influenza virus infection. Previous studies have indicated that the complement system plays an important role in viral clearance and regulation of inflammatory responses, which may contribute to the relatively low mortality observed even after infection with highly pathogenic influenza viruses [64]. By contrast, pigs are more prone to developing severe pneumonia following high-dose SIV infection or secondary bacterial co-infection, underscoring substantial interspecies differences in immune response magnitude and immunopathological outcomes.

On the basis of these pathological and immunological features, guinea pig models are particularly well suited for comparing the pathogenicity of influenza viruses from different host origins within a single host species and for investigating aerosol transmission efficiency and early innate immune responses. However, because pulmonary lesions are comparatively mild and the availability of immunological reagents remains limited, guinea pigs retain notable constraints in faithfully modelling the immune responses associated with natural SIV infection in pigs.

### 4.4. Pig Models: The Natural Host, Capturing Complete Pathogenesis and Immunological Complexity

As the natural host of SIV, pig models most closely reflect field outbreaks with respect to both pathology and immune responses. Following SIV infection, pigs can reliably develop acute respiratory disease dominated by pulmonary injury, and the core pathological features are highly consistent across different viral strains and experimental conditions. Gross examination typically reveals characteristic dark red to purple areas of pulmonary consolidation, distributed in a multifocal or coalescing pattern, most commonly involving the cranial and middle lung lobes. The severity of these lesions correlates closely with pulmonary viral replication levels and the clinical severity of disease.

**Figure 4:**
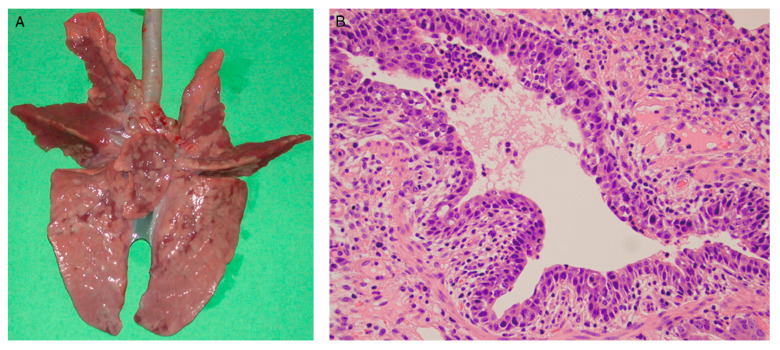
Gross and microscopic pneumonia in pigs following infection with an H1N1 swine influenza virus. (**A**) Gross lung lesions characterised by dark red to purple consolidation, predominantly affecting the cranial and middle lobes. (**B**) Necrotising bronchiolitis observed at 3 days post inoculation with SIV. Epithelial cells exhibited necrosis and reactive hyperplasia, with sloughed cells present within the airway lumen, which contained cellular debris, proteinaceous fluid, and small numbers of leukocytes.

Histopathological evaluation indicates that lesions are characterised primarily by necrotising bronchiolitis and bronchointerstitial pneumonia. Typical changes include degeneration, necrosis, and sloughing of bronchiolar epithelial cells, with cellular debris and proteinaceous exudates within the lumen, accompanied by prominent peribronchiolar lymphocytic cuffing. In some regions, varying degrees of alveolar collapse and interstitial inflammatory cell infiltration can be observed. Acute lesions usually peak at approximately 3–5 days post infection and subsequently begin to resolve, with progressive reparative changes characterised by regenerative hyperplasia of the bronchial epithelium and relative attenuation of inflammation. Overall, although acute pulmonary injury can be marked, the pathological process is generally self-limiting and constitutes a typical pathological spectrum of SIV infection in the porcine lung. Representative pathological findings reported in prior studies are shown in Figure 4, which summarises gross lesions and histological injury patterns in pigs following SIV infection [65].

From an immunological perspective, SIV infection induces a highly coordinated set of immune responses in the porcine lung, characterised by concomitant innate immune activation, enhancement of adaptive immunity, and engagement of immunoregulatory mechanisms. During the early phase of infection, multiple innate and pro-inflammatory mediators are markedly upregulated in lung tissues, including IFN-α, IL-6, IFN-γ, and IL-12. The levels of these cytokines correlate positively with pulmonary viral loads and the extent of tissue injury and are important drivers of acute inflammation and clinical manifestations. Against this background, adaptive immunity is rapidly activated. Flow cytometric analyses of mononuclear cells isolated from lung tissues, bronchoalveolar lavage fluid, and tracheobronchial lymph nodes at day 3 and day 6 after SIV infection have shown marked enrichment and activation of multiple immune cell subsets at sites of infection, including CD4^+^ and CD8^+^ T lymphocytes, cytotoxic T cells, γδ T cells, natural killer cells, and cell populations enriched for dendritic cells, suggesting that SIV infection can rapidly elicit T cell-mediated adaptive immune responses during the acute phase [66].

In parallel, antigen-specific IgA responses can be detected in lung tissue and bronchoalveolar lavage fluid, indicating an important contribution of mucosal humoral immunity to viral clearance. Notably, alongside heightened inflammation, immunoregulatory components are also upregulated, including increased proportions of FoxP3^+^ regulatory T cells and enhanced expression of anti-inflammatory cytokines such as IL-10 and TGF-β. These findings suggest that negative-feedback mechanisms are simultaneously engaged during antiviral responses. Collectively, these immunological features indicate that SIV infection in pigs does not simply induce uncontrolled inflammation but rather involves a dynamic balance between inflammatory injury and immunoregulation [67,68].

Under conditions where the vaccine strain and the challenge strain are antigenically mismatched, non-neutralising antibodies induced against heterologous viruses may exacerbate pulmonary inflammation upon re-exposure and even aggravate lesion severity. This phenomenon is defined as vaccine-associated enhanced respiratory disease (VAERD) [69,70]. The existence of VAERD indicates that vaccine evaluation in the pig model should not focus solely on protection rates and viral clearance but should also systematically assess potential immunopathological risks. Because pigs are the natural host of SIV, their respiratory immune architecture can faithfully reflect immune dysregulation under antigen mismatch conditions, giving this model unique value for identifying such risks.

However, despite the high biological relevance of the pig model, its outbred nature may introduce substantial inter-individual variability. The high polymorphism of the swine major histocompatibility complex (SLA) can influence antigen presentation and T cell response magnitude, thereby contributing to variability in vaccine responsiveness among animals [71]. Therefore, experimental design should minimise genetic heterogeneity through source standardisation, age matching, and appropriate stratified statistical analysis.

### 4.5. Non-Human Primate Models: Immunological Relevance and Ethical and Economic Constraints

**Figure 5:**
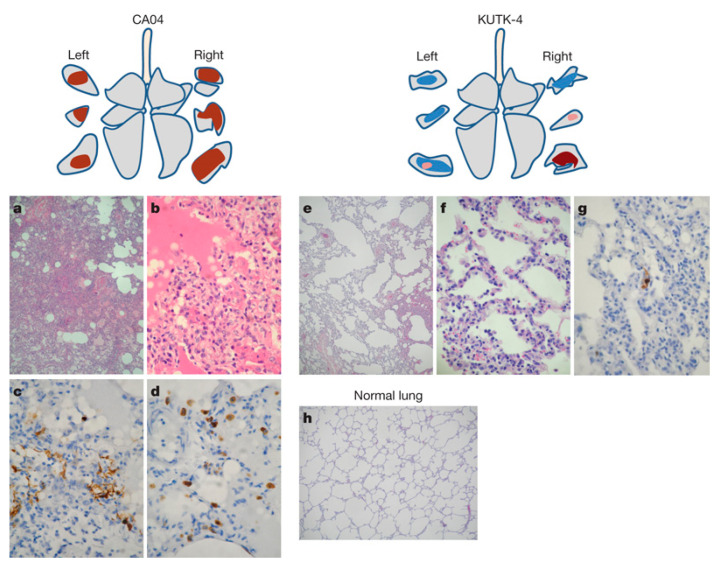
Representative pulmonary pathological findings and viral antigen distribution in cynomolgus macaques at 3 days post infection following inoculation with CA04 (A/California/04/2009 [H1N1]) or KUTK-4 (A/Kyoto/UTK-4/2009 [H1N1]). Panels (**a**–**d**) show CA04 infection (monkey 1), panels (**e**–**g**) show KUTK-4 infection (monkey 7), and panel (**h**) shows mock-infected controls, all at 3 dpi. One to two sections were examined from each lung lobe. Lesion distribution and antigen-positive areas are illustrated using schematic lung lobe diagrams combined with cross-sectional images. Colour coding indicates the following: brown, severe lesions with moderate to abundant antigen-positive cells; pink, mild lesions with few antigen-positive cells; blue, alveolar wall thickening with largely preserved air spaces. Magnifications: (**a**,**e**,**h**), ×40; (**b**–**d**,**f**,**g**), ×400.

Commonly used non-human primate models for influenza include rhesus macaques and cynomolgus macaques. Studies have shown that infection of non-human primates with the 2009 swine-origin H1N1 virus generally results in pulmonary lesions dominated by peribronchiolar inflammation and mild-to-moderate interstitial pneumonia, rather than extensive diffuse alveolar damage, indicating relatively moderate pathogenicity consistent with the clinical features observed in human infections, as illustrated in Figure 5 [72]. By contrast, in non-human primate models, highly pathogenic influenza viruses such as the 1918 H1N1 strain can induce severe respiratory disease characterised by pronounced diffuse alveolar damage, pulmonary oedema, and more severe inflammatory pathology, closely reflecting viral virulence [73,74].

At the level of immune responses, non-human primates exhibit strong similarities to humans and pigs in cellular immune repertoires, Fc receptor composition, and cytokine networks, conferring clear advantages in immunological relevance. Analyses of immune gene expression have demonstrated that sustained and intense innate immune activation during early infection—particularly marked upregulation of type I interferons and pro-inflammatory mediators—is strongly associated with severe pulmonary injury and poor outcomes, whereas more moderate inflammatory responses coupled with timely viral clearance are typically linked to milder clinical and pathological manifestations [75]. These patterns have been repeatedly confirmed in comparative studies of highly pathogenic versus low-pathogenic influenza viruses and are consistent with the immunopathological features observed in severe human influenza cases and severe SIV infections in pigs.

Overall, the principal value of non-human primate models lies in the validation of the safety and key immunological endpoints of high-risk candidate vaccines, monoclonal antibodies, or antiviral interventions, rather than in routine screening of SIV pathogenicity or large-scale immunogenicity assessments. Their high costs, stringent ethical constraints, and limited sample sizes make it impractical for these models to replace pigs as the central experimental system for studying natural SIV infection and population-level immune protection.

### 4.6. Cross-Model Comparative Analysis: Differences in Pathology, Viral Kinetics, and Immune Architecture

**Figure 6:**
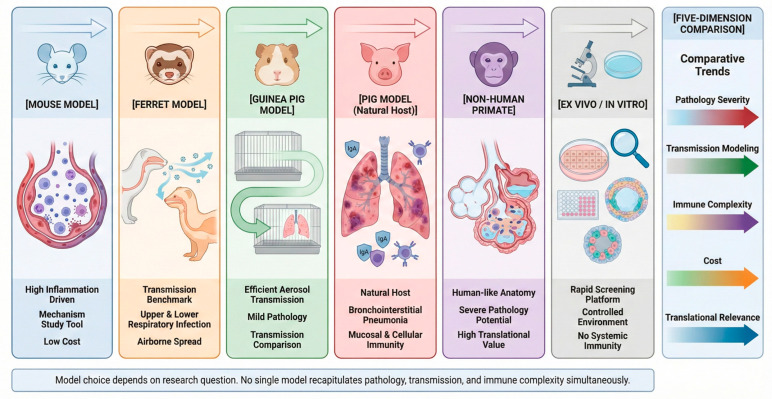
A comparative overview of major animal and ex vivo/in vitro models used in swine influenza virus (SIV) research. A schematic comparison of mice, ferrets, guinea pigs, pigs, non-human primates, and in vitro/ex vivo systems across key dimensions relevant to swine influenza virus (SIV) research, including pathological severity, transmission efficiency, immune complexity, cost, and translational relevance. The figure highlights the complementary roles of different models and underscores the necessity of tailoring multi-model strategies to specific research questions.

Although each animal model provides unique value in SIV research, interpretation of their results must be grounded in systematic cross-model comparisons across key biological dimensions rather than isolated, model-specific descriptions, with detailed comparisons presented in Figure 6 and Table 2.

In terms of pathological severity, clear hierarchical differences are observed among species, and these patterns are influenced by viral subtype and virulence. The pig model most faithfully reproduces the characteristic necrotizing bronchiolitis and bronchointerstitial pneumonia seen in natural infection, with lesion distribution highly consistent with field cases in swine herds. In the ferret model, pathological changes show subtype-dependent variation: highly pathogenic strains can induce pronounced lower respiratory tract inflammation, whereas seasonal-like strains are more often confined to the upper respiratory tract. Mice commonly exhibit diffuse inflammatory infiltration; however, the lesion distribution does not fully match that of pigs, particularly with respect to bronchiolar necrosis and alveolar structural damage. Guinea pigs typically develop only mild bronchointerstitial changes with limited tissue destruction. The pathological severity observed in non-human primates generally falls between that of pigs and ferrets and is closely associated with viral virulence and inoculation dose.

Regarding viral replication kinetics, systematic differences also exist among models, and the timing of peak replication and viral load is influenced by viral strain, inoculation dose, and infection route. In pigs, the replication curve closely mirrors the timeline of field infection, showing a well-defined viral peak and a predictable clearance phase, and a stable shedding window can usually be detected in nasal swabs. Ferrets support highly efficient upper respiratory replication and exhibit stable aerosol transmission, making them a key model for studying transmission dynamics. Mice typically require host-adapted viral strains to achieve efficient replication, and the replication capacity of different swine-origin H1N1 isolates varies substantially in this model. Although guinea pigs support viral transmission, the correlation between clinical signs and viral load is relatively weak, making it difficult to infer true replication intensity from clinical presentation alone. Non-human primate models display replication efficiency and clearance kinetics that approximate human infection, but their experimental scalability is limited.

At the level of immune architecture, the differences among models are even more pronounced. The pig model can simultaneously reproduce mucosal IgA responses, CD4^+^/CD8^+^ T cell activation, γδ T cell participation, and immunoregulatory feedback mechanisms, thereby forming a relatively complete respiratory mucosal immune network. Ferrets exhibit human-like antibody kinetics and immune imprinting phenomena, but the depth of cellular immune characterisation remains limited. Mice are well suited for dissecting innate signalling pathways, inflammatory cascades, and cytokine networks; however, the complexity of their respiratory mucosal immune structure is lower than that of pigs. In guinea pigs, the relative scarcity of species-specific immunological reagents restricts fine-scale immune phenotyping. Non-human primates possess high translational relevance in immune architecture, allowing for observation of tissue-resident T cell formation and long-term immune memory; however, ethical and cost constraints limit their large-scale application.

Although a tiered, multi-model collaborative framework offers clear theoretical advantages, its practical implementation must be aligned with specific research objectives. Taking the evaluation of a hypothetical universal influenza vaccine candidate as an example, a stepwise experimental pathway can be constructed. First, at the in vitro stage, MDCK cells can be used for rapid screening of viral replication inhibition and preliminary antigen stability assessment, enabling early elimination of candidates with insufficient replication control or unstable antigen conformation [76]. Subsequently, the mouse model can be employed to dissect vaccine-induced innate immune activation, including interferon signalling, inflammatory cytokine profiles, and early CD8^+^ T cell expansion, thereby establishing a mechanistic immunogenicity foundation [77,78]. Building on these findings, the ferret model can be used to evaluate the vaccine’s ability to block aerosol transmission and shorten the viral shedding window, thereby assessing its potential population-level impact [79]. Finally, protective efficacy, safety, and durability of immunity should be validated in pigs to confirm real-world translational relevance in the natural host [79]. This tiered strategy progressively strengthens the evidence base while controlling cost and risk and reduces interpretive bias associated with reliance on a single model.

Taken together, these dimensions demonstrate that the various models exhibit complementary characteristics in pathological distribution, viral replication patterns, transmission efficiency, and immune complexity. No single model can fully capture the complete biological spectrum of natural host infection. Therefore, a multi-model integration strategy is not merely a methodological supplement but a necessary biological framework for reducing interpretive bias and improving the translational reliability of SIV vaccine evaluation.

## 5. Current Challenges and Future Perspectives

### 5.1. Major Challenges in Current Research

(1) Model-specific differences drive variability in validation outcomes

Existing animal models differ intrinsically from target hosts with respect to infection routes, receptor distribution, and immune composition. Immune response magnitudes vary not only among species but also among strains within the same model. Such interspecies and inter-individual variability complicates direct comparison of vaccine or intervention performance across models and limits extrapolation to target animals or field conditions.

(2) Biosafety constraints restrict critical validation studies

Research involving highly pathogenic or emerging agents typically requires biosafety level 3 (BSL-3) or higher containment. The availability, geographic distribution, and operational costs of high-containment facilities impose practical limitations, which can prolong study timelines and restrict systematic evaluation of certain models and strategies.

(3) Cost and ethical considerations limit large-animal studies

Large-animal models offer advantages in approximating natural infection and immune responses; however, high husbandry and experimental costs, stringent ethical review requirements, and limited sample sizes render them unsuitable for early-stage screening or large-scale validation. By contrast, murine models are economically efficient but possess simplified immune architectures that cannot fully substitute for target hosts.

(4) Natural infection processes remain incompletely reproduced

Marked differences among species in tissue tropism, immune regulation, and disease progression mean that most existing systems remain approximations and cannot comprehensively mimic natural infection or complex immunological outcomes. This limitation underscores the difficulty of relying on any single model as a sufficient predictor of vaccine performance.

### 5.2. Future Directions and Priorities

Future development of animal models is expected to shift from descriptive characterisation of infection outcomes toward finer-resolution analyses of immune processes and tissue microenvironments. Integration of multi-omics technologies, advanced in vitro platforms (such as organoids and organ-on-chip systems), and computational immunology approaches may partially mitigate the limitations of in vivo models. A more practical strategy is the establishment of tiered, multi-model evaluation frameworks: murine systems for early screening, intermediate-sized animals for calibration of immune response features, and eventual validation in target hosts. Coupled with mechanism-oriented integration of immunological endpoints, such approaches may reduce uncertainty arising from model differences and enhance the predictability and reproducibility of vaccine development pipelines.

## 6. Conclusions

Overall, the infection and immune processes of swine influenza virus (SIV) exhibit marked host dependence. Differences among animal models in patterns of pathological injury, immune response architecture, and transmission characteristics determine their functional positioning within the research framework. This host dependence is reflected not only in histopathological lesion distribution but also in key immunological dimensions, including thresholds of innate immune activation, the integrity of mucosal immune structures, and the capacity to generate tissue-resident T cells.

The mouse model offers clear advantages for mechanistic dissection and early-stage screening; however, its immune architecture deviates from that of the natural host. Ferret and guinea pig models are more suitable for studying transmission dynamics and early infection events, but they provide limited fidelity in recapitulating the natural disease course and immune complexity of swine influenza. In contrast, the pig model most comprehensively reproduces pulmonary pathology and the coordinated innate–adaptive immune responses observed in the natural host, making it an indispensable core system for vaccine efficacy evaluation and immunological mechanism studies. It also serves as a critical platform for identifying potential immunopathological risks under antigen mismatch conditions. Non-human primate models are primarily used for safety and immunological validation of high-risk strains or intervention strategies and provide complementary value in cross-species risk assessment and translational research.

Therefore, no single “universal model” represents an optimal solution for SIV research. The differences among models should not be viewed as limitations but rather as reflections of the biological diversity inherent in virus–host interactions across species. According to the research stage and specific scientific questions, a hierarchical, multi-model coordinated strategy can reduce bias arising from model-specific constraints and improve the interpretability and translational relevance of vaccine evaluation. This mechanism-oriented, target host-anchored model selection framework is likely to represent a more robust path for future SIV vaccine development and pathogenesis research, while also providing an experimental basis for risk identification and forward-looking assessment of emerging swine-origin reassortant strains.

## Figures and Tables

**Table 1:** Analysis of the Advantages and Limitations of Different Animal Models.

Animal Model	Core Strengths	Major Limitations	Availability of Immunological Reagents	Main Research Applications
Mouse	1. Low cost, short generation time, suitable for large-scale experiments. 2. Powerful genetic manipulation tools enabling immunological and transcriptomic analyses. 3. Amenable to construction of specific immune-deficient strains.	1. Common laboratory strains (e.g., C57BL/6, BALB/c) are not naturally susceptible to human or swine influenza viruses. 2. Respiratory epithelium predominantly expresses α-2,3–linked sialic acid receptors. 3. Simplified transmission patterns and poor support for aerosol spread.	1. Species-specific antibodies, cytokine detection kits, and flow cytometry markers are extremely abundant. 2. Mature resources are available for single-cell sequencing, transgenic reporter systems, and immunodeficient strain. 3. Supports multiparameter flow cytometry, spatial transcriptomics, and immune repertoire analysis.	1. Identification of viral pathogenic determinants. 2. Preclinical vaccine testing.
Ferret	1. No viral adaptation usually required; efficient replication in the respiratory tract. 2. Respiratory tract anatomy and receptor distribution similar to humans. 3. Supports droplet and aerosol transmission, mimicking natural spread. 4. Relatively compact genome facilitates development of specific reagents.	1. Differences from pigs and incomplete recapitulation of all transmission routes. 2. High cost and limited availability restrict large-scale studies. 3. Highly pathogenic strains (e.g., H5N1) can cause severe disease, complicating longitudinal observation.	1. Species-specific immunological antibodies and cytokine detection reagents are relatively limited. 2. Some human- or canine-derived antibodies show cross-reactivity, but their stability is insufficient. 3. Multiparameter flow cytometry and fine-scale immune cell subset profiling are constrained.	1. Transmission mechanisms. 2. Modelling influenza-like disease in humans. 3. Evaluation of transmission-blocking interventions.
Guinea pig	1. Highly susceptible to certain influenza viruses (e.g., seasonal H3N2 strains). 2. Efficient transmission via direct contact and aerosols. 3. Relatively easy husbandry and material availability.	1. Limited overt clinical signs and mild lung pathology.	1. Dedicated immunological antibodies and cytokine detection reagents are severely lacking. 2. Flow cytometric cell subset analysis and immune phenotyping capabilities are limited. 3. Studies often rely on transcriptional-level analyses to substitute for protein-level detection.	1. Studies of viral transmission dynamics.
Pig	1. Natural host of SIV with clinical manifestations closely resembling field infections. 2. Critical “mixing vessel” for reassortment between avian and human influenza viruses. 3. Expression of both α-2,3 and α-2,6 sialic acid receptors. 4. Physiological, anatomical, and immunological features similar to humans.	1. Immune correlates of protection remain incompletely defined. 2. High husbandry costs and long experimental duration. 3. Substantial inter-individual variability. 4. Operational complexity and genetic heterogeneity may confound outcomes.	1. Species-specific monoclonal antibodies and flow cytometry reagents are gradually increasing. 2. Relatively mature immune cell profiling systems (e.g., CD4, CD8, and B cells) have been established. 3. Compared with mice, reagent diversity remains limited and costs are higher.	1. Viral replication in the natural host. 2. Adaptation prior to cross-species transmission. 3. Vaccine evaluation.
Non-human primate	1. Closest phylogenetic relationship to humans, with highly similar immune systems and organ structures. 2. Able to sustain infection with highly pathogenic influenza viruses and allow for longitudinal monitoring.	1. High cost and limited scalability. 2. Complex ethical considerations. 3. Transmission mechanisms incompletely characterised.	1. Highly cross-compatible with humans, allowing for direct use of many human-derived antibodies. 2. Supports multiparameter flow cytometry, single-cell omics, and spatial immune analysis. 3. However, experimental scale is restricted by ethical considerations and cost.	1. Durability of vaccine-induced immunity. 2. Mechanisms of highly pathogenic SIV infection.
Ex vivo/in vitro systems	1. Primary porcine respiratory epithelial cells and lung slices recapitulate early cellular and tissue-level infection dynamics. 2. Cell lines (e.g., MDCK, PK-15) enable rapid screening of antivirals and replication kinetics.	1. Cannot substitute for whole-animal immune responses or transmission studies.	1. Primary porcine cells can use some porcine antibodies and cytokine detection reagents, but the range remains limited. 2. Cell lines (e.g., MDCK, PK-15) are mainly used for viral replication studies and have limited immune phenotyping capacity. 3. Transcriptomics and proteomics can be integrated to analyse immune signalling pathways, but a complete immune cell system is lacking.	1. Virus–host interaction studies. 2. Antiviral compound screening.

**Table 2:** Cross-model comparison of pathological features, viral kinetics, and immune characteristics in SIV research. This table was compiled based on publicly reported experimental infection data from [32,39,40,41,42,43,44,45,46,47,48,49,50,51,52,53,54,55,56,57,58,59,60,61,62,63,64,65,66,67,68,69,70,71,72,73,74,75]. The severity of pathology, peak viral replication time, and magnitude of immune responses across different animal models are influenced by multiple factors, including viral subtype, strain background, inoculation dose, and infection route. Therefore, the values presented here reflect typical observations reported in representative studies rather than absolute measurements or universal patterns. The time to peak viral replication is listed only when explicitly reported in the original studies; parameters not systematically described in the literature were not inferred. Owing to differences in experimental design, data across studies are not fully comparable. The purpose of this table is to provide a structural cross-model comparison framework rather than a direct quantitative ranking of model performance.

	Mouse	Ferret	Guinea Pig	Pig	Non-Human Primate Model
Viral replication characteristics	Most swine-origin H1N1 and recombinant viruses can replicate; some require adaptive mutations.	H3N2 mainly restricted to the nasal cavity; pH1N1 replicates throughout the respiratory tract; H5N1 shows a lower respiratory tract preference.	Susceptible to human-, swine-, and avian-origin viruses.	Stable replication; temporal kinetics closely match natural outbreaks.	Productive replication; virulence is strain-dependent.
Peak time of viral replication	2–3 dpi (some strains show obvious lesions at 3–5 dpi)	1–4 dpi (strain-dependent)	Histological changes detectable at 3–5 dpi	Pathological peak typically at 3–5 dpi	Apparent lesions around 3 dpi (in some strains)
Viral shedding duration	Relatively short (declines by 5–7 dpi)	Prominent nasal shedding; moderate duration	Detectable shedding; weak correlation with clinical signs	Shedding window is predictable	Moderate; rarely used for transmission studies
Major pulmonary pathological changes	Acute bronchitis/bronchiolitis plus acute interstitial pneumonia; epithelial injury and desquamation	H3N2: mild upper respiratory damage pH1N1: bronchopneumonia H5N1: severe alveolitis	Bronchointerstitial pneumonia; mild alveolar epithelial degeneration	Necrotizing bronchiolitis; bronchointerstitial pneumonia	Mild–moderate peribronchiolar inflammation and interstitial pneumonia
Histopathological features	Alveolar septal thickening; neutrophil and macrophage infiltration; pulmonary oedema/haemorrhage/atelectasis	Lesion distribution highly correlates with viral antigen; H5N1 may cause necrosis and haemorrhage	Interstitial oedema; lymphocyte/histiocyte infiltration; mild congestion	Epithelial necrosis and desquamation; peribronchiolar lymphoid cuffing; alveolar collapse	Mild lesions in low-virulence strains; severe diffuse alveolar damage with highly virulent strains
Severe disease features	Highly virulent strains may cause necrotizing bronchiolitis plus severe alveolitis	H5N1 causes severe lower respiratory tract injury	Extensive haemorrhagic pneumonia is uncommon	High dose or secondary infection may progress to severe pneumonia	Highly virulent strains (e.g., 1918) may induce diffuse alveolar damage
Overall pathology severity	Moderate (strain-dependent)	H3N2: low; pH1N1: moderate; H5N1: high	Low–moderate	Moderate–high (self-limiting)	Low–high (virulence dependent)
Transmission pattern	Does not reliably support aerosol transmission	Robust aerosol and contact transmission model	Classical aerosol transmission model	Suitable for herd transmission observation	Rarely used for transmission studies
Consistency with natural swine infection	Differences exist in lesion distribution and shedding pattern	Respiratory distribution resembles humans	Mild disease; difficult to fully mimic severe swine infection	Highly consistent with natural infection	Immune responses highly similar to humans
Innate immune features	Strong upregulation of type I IFN, TNF-α, MIP-1α/MIP-2; pronounced inflammatory amplification	IFN-γ-associated cellular responses active	Complement system involved in antiviral defence	IFN-α, IL-6, IL-12 upregulated	Sustained type I IFN responses associated with disease severity
Early cellular infiltration	Neutrophils and macrophages dominate	Prominent respiratory inflammatory cell infiltration (strain-dependent)	Mainly lymphocytes and histiocytes	Enrichment of CD4^+^, CD8^+^, γδT, and NK cells	Immune cell spectrum closely resembles humans
Adaptive immunity	CD8 T cells involved in mid–late phase; IL-10 participates in negative feedback	HI and neutralising antibodies detectable ~1 week post infection	Antibody responses reported but limited immunotyping	T cell activation detectable as early as 3–6 dpi	Cellular immune responses highly similar to humans
Mucosal immune architecture	IgA present but structurally limited	Antibody kinetics similar to humans	Limited data available	Prominent IgA responses in BAL fluid	Mucosal immune structure close to humans
γδ T cells	Rare	Not systematically reported	Not systematically reported	Clearly involved	Present at low frequency
Cross-protection	Moderate	Strongly influenced by prior exposure	Unclear	Antigen mismatch may lead to VAERD	Mainly used for safety validation

## Data Availability

No new data were created or analyzed in this study.
